# Guillain-Barré Syndrome as a Complication of COVID-19

**DOI:** 10.7759/cureus.12695

**Published:** 2021-01-14

**Authors:** Adeel S Zubair, Aarij S Zubair, Kunal Desai, Ahmad Abulaban, Bhaskar Roy

**Affiliations:** 1 Neurology, Yale University, New Haven, USA; 2 Neurology, St. John's University, New York, USA

**Keywords:** guillain-barré syndrome, neuromuscular disorder, covid-19, coronavirus disease 19, neurology, critical illness polyneuropathy, critical illness myopathy

## Abstract

Coronavirus disease 2019 (COVID-19) is associated with multiple neurological complications including Guillain-Barre syndrome (GBS).^ ^While there are reports of COVID-19 -related GBS cases, much remain unknown. We report two cases of GBS-associated COVID-19, which started about eight weeks after the initial COVID-19 infection. Such a long duration between infection and symptom onset of GBS is unusual for post-infectious GBS. Moreover, severely ill patients with COVID-19 may have prolonged hospital stay leading to critical illness myoneuropathy. Diagnosing superimposed GBS can be challenging in such cases. Clinical suspicion, nerve conduction studies with electromyography, and cerebrospinal fluid analysis can help in making the correct diagnosis. Both presented cases responded to intravenous immunoglobulin therapy.

## Introduction

In December of 2019, a novel coronavirus, SARS-CoV-2, emerged, infecting millions of people worldwide and causing significant morbidity and mortality to the affected individuals [1]. Coronavirus disease 2019 (COVID-19) is associated with various neurological complications, including Guillain-Barré syndrome (GBS) [1-4].

GBS is a rare autoimmune inflammatory neuropathy with a varied clinical spectrum [5]. The classic form of GBS, acute inflammatory demyelinating polyneuropathy (AIDP), presents with ascending weakness, sensory deficits, and loss of deep tendon reflexes [5,6]. It can also present as pure motor, pure sensory, and pharyngeal-cervical-brachial variants, along with Miller Fisher syndrome (MFS) with cranial nerve involvement [5,6]. Electrophysiologic studies can further characterize GBS as AIDP, acute motor axonal neuropathy (AMAN), and acute motor sensory axonal neuropathy (AMSAN) [5,6].

Previous reports have shown that several viral (cytomegalovirus, Epstein-Barr virus, Hepatitis E virus, and Zika virus) and bacterial (campylobacter jejuni and mycoplasma pneumoniae) infections can trigger an aberrant immune response attacking the peripheral nerves, leading to GBS [5,6]. Additionally, GBS has been reported in patients with the Middle East respiratory syndrome (MERS) [7]. While there are some case reports and case series with COVID-19-related GBS, there is a knowledge gap and the entire clinical spectrum of COVID-19-related GBS remains unknown [1,8]. Here we report two cases of GBS associated with COVID-19 to highlight some critical clinical features and diagnostic challenges.

## Case presentation

Case 1

A 32-year-old man with no relevant medical history presented with hypoxia, tachypnea, and fever from COVID-19 which was confirmed by RNA PCR test. He was admitted to the medical ICU and developed acute respiratory distress syndrome (ARDS). He received tocilizumab (single dose), hydroxychloroquine (three-day course), and remdesivir (100mg 14-day course). His ventilation requirements continued to increase and ultimately, he required veno-venous extracorporeal membrane oxygenation. He spent 60 days in the ICU, which was further complicated by a gastrointestinal bleed. 

**Figure 1:**
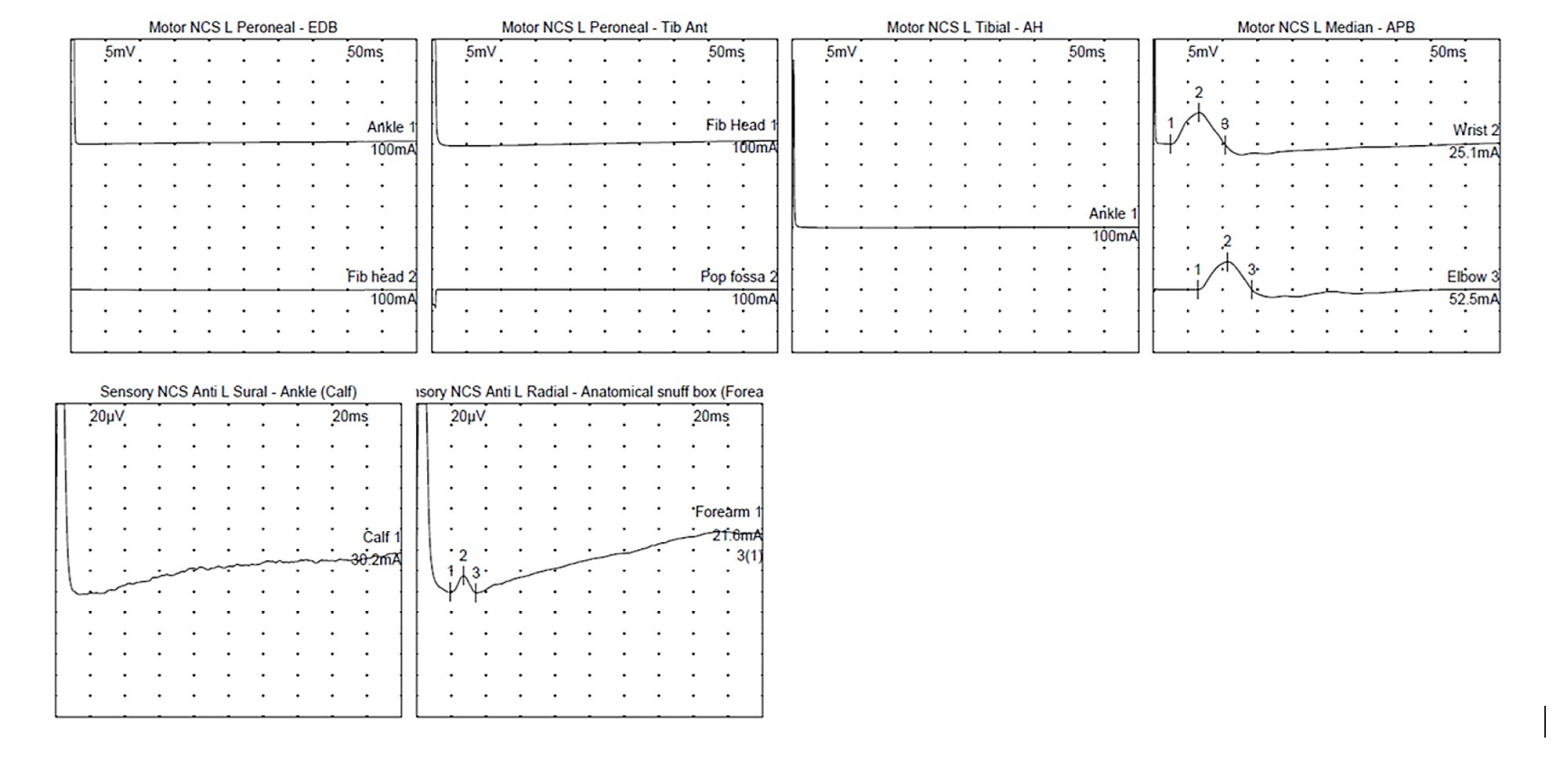
Nerve conduction studies (NCSs) waveforms for patient 1. Motor responses of the left peroneal nerve (recording from extensor digitorum brevis and tibialis anterior) and tibial nerve (recording from abductor hallucis) were absent. Motor response of the left median nerve was normal. Sensory response of the left sural nerve was absent. Sensory NCS of the left radial nerve was borderline normal. [Of note, this was a bedside study].

After the prolonged ICU stay, he was deconditioned and had a significant generalized weakness. He began to work with physical therapy and was able to walk with an assistive device. Over the next five days, he noted paresthesia in his lower extremities along with a progression in weakness, eventually requiring two-person assist to ambulate. Neurological examination revealed atrophy of anterior and posterior compartment leg muscles and 1/5 strength in ankle dorsi and plantarflexion. Proximal strength was preserved, and he did not have any facial weakness. Sensation to light touch was intact and vibration sensation was mildly decreased. Reflexes were 2+ at upper extremities, brisk at the patella bilaterally, and absent at the ankles. While there was a concern for a critical illness myoneuropathy, the clinical presentation, including lack of proximal weakness, sudden deterioration in motor strength, and new onset sensory neuropathy while recovering were atypical [9-11]. Electromyography (EMG) and nerve conduction studies (NCSs) showed severe axonal sensorimotor polyneuropathy with ongoing denervation changes consistent with a diagnosis of acute motor sensory axonal neuropathy, a variant of GBS (Figure 1). Comprehensive workup revealed normal B1, B6, B12, ANA <1:80, negative for Hepatitis B and HIV, normal SPEP, UPEP, negative for GM1 and GD1a/b antibodies. CSF analysis showed albumino-cytological dissociation with four nucleated cells and protein of 127.6 mg/dL, further confirming the diagnosis.

He responded to intravenous immunoglobulin (IVIg) therapy. Prior to discharge from the hospital, he was able to stand up with assistance. He was discharged to an acute rehabilitation center where he continued to improve and was able to walk with a walker with exam notable for improved strength in dorsi and plantarflexion (2/5).

Case 2

**Figure 2:**
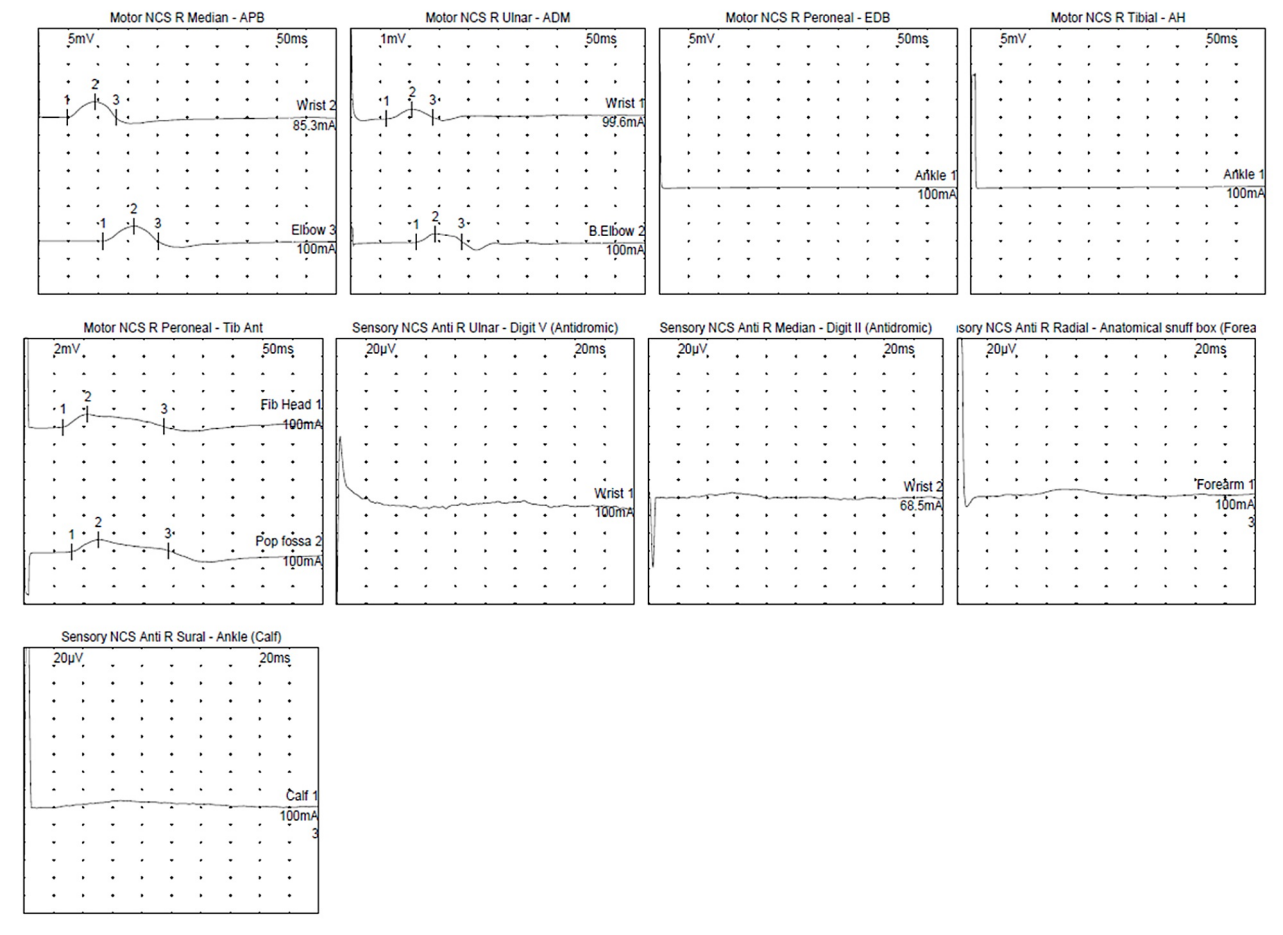
Nerve conduction studies (NCSs) waveforms for patient 2. Motor responses of the right peroneal nerve (recording from extensor digitorum brevis) and tibial nerve (recording from abductor hallucis) were absent. Motor response of the left peroneal nerve recording from tibialis anterior showed reduced response amplitude. Motor NCSs of the right median and ulnar nerves showed prolonged distal latencies and slowing of conduction velocities. Response amplitudes were reduced for right ulnar nerve. Sensory responses of the right sural, median, ulnar and radial nerves were absent.

A 61-year-old man with known diabetes with no pre-existing neuropathy, severe lumbar stenosis (L4-L5), and right foot drop at baseline, presented with two days of generalized weakness and diarrhea and was positive for COVID-19 (RNA PCR). Shortly after admission, he went into acute respiratory failure requiring intubation. He also received a dose of tocilizumab for COVID-19 after intubation. He had a prolonged ICU course of over seven weeks which was complicated by aspiration pneumonia, urinary tract infection, and critical illness myoneuropathy. He was extubated and his medical issues stabilized. He was discharged to acute rehabilitation facility 60 days after initial admission but there he developed new-onset leg weakness, gait instability, and soon after his hands were involved. He was readmitted with increasing weakness. Neurological examination revealed atrophy of intrinsic hand and foot muscles. He had mild proximal upper extremity weakness, with 2/5 strength in ankle dorsi and plantarflexion. Vibration sensation was absent at the great toes and patella, and he was areflexic. His EMG/NCSs showed a severe axonal sensorimotor polyneuropathy (Figure 2). CSF analysis showed no nucleated cell and mild elevation of protein to 54 mg/dL (normal: 15-45 mg/dl). He also was negative for Hepatitis B in addition to HIV and had a normal SPEP, normal vitamin B12, and no ganglioside antibodies. He responded to a course of IVIg therapy with improvement in his strength, with improvement in proximal upper extremity strength (4+) as well as distal strength (improvement in ankle strength). He was eventually discharged to a rehabilitation where he continued therapy for improving his strength (Table 1).

**Table 1:** Pertinent laboratory workup for the presented cases. NP: not performed; CSF: cerebrospinal fluid.

	Patient 1	Patient 2
Antinuclear antibody (ANA)	Negative	NP
Serum protein electrophoresis (SPEP)	Negative	Negative
Erythrocyte sedimentation rate (ESR) [0-15 mmg/hr]	45	120
Glucose A1c [normal <5.7]	6.3	5.3
Vitamin B12 [220-960 pg/mL]	924	729
Vitamin B1 [8-30 nmol/L]	16	NP
Vitamin B6 [2.1-21.7 ng/mL]	7.5	NP
Lyme antibody	NP	Negative
Campylobacter Jejuni antibody titer [ref <0.9]	0.94	NP
Human immunodeficiency virus (HIV)	Negative	Negative
Hepatitis B and C	Negative	Negative
GM1, GD1a/b gangliosides	Negative	Negative
Paraneoplastic panel	NP	Negative
CSF analysis		
RBC	11	0
Nucleated cells	2	0
Glucose [40-75 mg/dl]	85	85
Protein [15-45 mg/dl]	127.6	54

## Discussion

GBS can be associated with COVID-19 [1-4,8,9]. Many patients with GBS present with an antecedent infection. The majority of GBS cases associated with COVID-19 had typical AIDP pattern, consisting of a sensorimotor, primarily demyelinating GBS. AMSAN variants are relatively rare (<15% of all reported cases). Most patients present with symptoms roughly nine days after onset of COVID-19 [1,8]. However, our patients developed GBS about eight weeks after the initial symptoms of COVID-19, which is atypical. Moreover, both of them were affected by critical illness myopathy/neuropathy, further making the diagnosis challenging. Interestingly, one of the patients retained reflexes which is unusual for GBS but can be seen in a small percentage of patients [5,6].

It is important to differentiate the causes of acute to subacute new-onset weakness in patients with COVID-19. In severe cases of the disease, patients may have prolonged hospital courses with significant intensive care unit stays. This can result in critical illness myopathy (CIM) and critical illness polyneuropathy (CIPN), and these conditions may present together. The pathophysiology of CIM is thought to be secondary to an inflammatory cascade due to microvascular, metabolic, and electrical alterations with atrophy due to increase muscle proteolysis with decrease in muscle protein synthesis [10]. Creatinine kinase can be elevated in these patients, and they often present with symmetric proximal muscle weakness which was not seen in these cases. CIPN can present with diminished reflexes, weakness, or paresthesia. Systemic inflammatory response syndrome is a frequent underlying factor for CIPN [11]. The pathophysiology of CIPN is likely from injury to the microcirculation of distal nerves, resulting in ischemia and axonal degeneration. CSF analysis in CIPN usually does not show albumino-cytological dissociation. 

Differentiating between CIPN/CIM and GBS can be nuanced and complicated, and as discussed above, CSF studies and EMG/NCS can be used to provide diagnostic evidence. However, sometimes, NCS/EMG alone may not be able to confidently differentiate between CIPN and axonal variant of GBS [5,11-14]. In a small percentage of cases reflexes can be intact in axonal variants of GBS. In such cases, a critical review of the clinical presentation, and CSF analysis can be helpful [12-14]. It is critically important to try and parse out these two diagnoses as there are treatment options available for GBS which can lead to improved clinical outcomes. At this point, there is no approved therapy for CIPN/CIM [12-14].

## Conclusions

GBS associated with COVID-19 can have a delayed onset. These cases are unique to currently published literature in their delayed clinical presentation. Moreover, in critically ill patients from COVID-19 with CIM/CIPN, diagnosis of GBS can be challenging. Clinical suspicion along with thorough EMG/NCSs and CSF analysis can help in making the correct diagnosis and initiation of appropriate intervention. With the cases of patients infected with SARS-COV-2 rising, it is prudent to remain vigilant to assess for possible treatable causes of neurological deficits in patients. While conditions such as CIM/CIPN have no current treatment, GBS, including atypical versions, can be treated with improvement in clinical outcomes. 
